# The plasma membrane-localized OsNIP1;2 mediates internal aluminum detoxification in rice

**DOI:** 10.3389/fpls.2022.970270

**Published:** 2022-09-12

**Authors:** Yuqi Wang, Shaohua Yang, Chune Li, Taijiao Hu, Siyu Hou, Qing Bai, Xiyue Ji, Feng Xu, Chongdai Guo, Min Huang, Yanfei Cai, Jiping Liu

**Affiliations:** ^1^Key Laboratory for Water Quality and Conservation of the Pearl River Delta, Ministry of Education, School of Environmental Science and Engineering, Guangzhou University, Guangzhou, China; ^2^Robert W. Holley Center, United States Department of Agriculture, Agricultural Research Service, Cornell University, Ithaca, NY, United States; ^3^Institute of Biotechnology, Fujian Academy of Agricultural Sciences, Fuzhou, China; ^4^School of Agriculture, Shanxi Agricultural University, Jinzhong, China; ^5^College of Natural Resources and Environment, South China Agricultural University, Guangzhou, China; ^6^Plant Breeding and Genetics Section, School of Integrative Plant Sciences, Cornell University, Ithaca, NY, United States

**Keywords:** aluminum tolerance, aquaporin, internal detoxification, nodulin 26-like intrinsic proteins, rice, external detoxification

## Abstract

Aluminum (Al) toxicity significantly restricts crop production on acidic soils. Although rice is highly resistant to Al stress, the underlying resistant mechanisms are not fully understood. Here, we characterized the function of OsNIP1;2, a plasma membrane-localized nodulin 26-like intrinsic protein (NIP) in rice. Aluminum stress specifically and quickly induced *OsNIP1;2* expression in the root. Functional mutations of OsNIP1;2 in two independent rice lines led to significantly enhanced sensitivity to Al but not other metals. Moreover, the *Osnip1;2* mutants had considerably more Al accumulated in the root cell wall but less in the cytosol than the wild-type rice. In addition, compared with the wild-type rice plants, the *Osnip1;2* mutants contained more Al in the root but less in the shoot. When expressed in yeast, OsNIP1;2 led to enhanced Al accumulation in the cells and enhanced sensitivity to Al stress, suggesting that OsNIP1;2 facilitated Al uptake in yeast. These results suggest that OsNIP1;2 confers internal Al detoxification *via* taking out the root cell wall’s Al, sequestering it to the root cell’s vacuole, and re-distributing it to the above-ground tissues.

## Highlights

- OsNIP1;2 facilitates internal aluminum (Al) detoxification in rice.
- *OsNIP1;2* expression is specifically induced by Al.
- OsNIP1;2 is critical for resistance to Al toxicity in rice.

## Introduction

Aluminum (Al) is prevalent in the earth’s crust (Liu et al., 2014; Kochian et al., 2015). Al has no known biological function, but the Al^3+^ ions released from acid soils are highly harmful to plants. Al stress restricts root growth, inhibits nutrient uptake from the root, and causes severe yield losses for crops grown on acid soils (Liu et al., 2014; Kochian et al., 2015; Singh et al., 2017). Moreover, the problem of Al toxicity on crop plants has been intensified worldwide by heavy applications of acid-forming nitrogenous fertilizers (Guo et al., 2010) and the frequent deposition of acid rain caused by deteriorating environments due to global climate changes. Although neutralizing soil acidity by liming could alleviate Al toxicity to plants, the process is costly, time-consuming, and less effective (Sade et al., 2016). Therefore, exploring the potential of plants to cope with Al stress is a practical and feasible approach to fighting against Al toxicity (Yuan et al., 2011).

Plants use several resistance mechanisms to withstand Al stress, including the external (exclusion) and the internal tolerance mechanisms. For example, many plants release organic acids from the root upon exposure to Al^3+^ ions. The released organic acids chelate the toxic Al^3+^ ions and thus decrease Al toxicity in the rhizosphere, the most commonly used exclusion mechanism in plants. In contrast, the internal tolerance mechanism involves detoxifying Al toxicity in the plant *via* closeting Al in the vacuole of the root cell and/or transporting it from the vulnerable root to the less-sensitive shoot tissues (Liu et al., 2014; Kochian et al., 2015).

Recent research has confirmed the cell walls in the root apical region as one of the most vulnerable targets for Al toxicity in plants (Sivaguru and Horst, 1998; Ma et al., 2004; Jones et al., 2006; Sivaguru et al., 2013). Therefore, decreasing Al accumulation in the cell wall of the root apex is critical for plants’ resistance to Al toxicity (Xia et al., 2010; Famoso et al., 2011; Wang et al., 2017).

Altering or modifying root cell-wall components could prevent Al accumulation in the root cell wall (Yang et al., 2008, 2011; Zhu et al., 2012). Another means to decrease the root-cell-wall Al content is to transport the Al to the cytosol through plasma-membrane (PM)-localized transporters. For instance, the PM-localized Nrat1 (Nramp aluminum transporter 1) facilitates the transport of Al in the root cell wall to the cytosol (Xia et al., 2010), playing a critical role in Al resistance in rice (Xia et al., 2010; Li et al., 2014). Moreover, we recently demonstrated that AtNIP1;2, an aquaporin (AQP) of the nodulin 26-like intrinsic protein (NIP) subfamily, is a critical component for Al resistance in Arabidopsis. AtNIP1;2 mediates moving the root-cell-wall Al to the root cytosol and subsequent Al translocation to the above-ground tissues (Wang et al., 2017, 2018, 2020, 2021).

Here, we further investigated the involvement of the NIP subfamily members in Al detoxification and resistance in rice. We report that OsNIP1;2, the closest homolog of AtNIP1;2, facilitates lowering the root-cell-wall Al concentrations and promotes root-to-shoot Al translocation. Furthermore, functional mutations of OsNIP1;2 sensitized the transgenic rice plants to Al toxicity. In conclusion, OsNIP1;2 is vital for Al resistance and detoxification in rice.

## Materials and methods

### Phylogenetic analysis

The ClustalW method with the MEGA 6.06 software was used to align the AtNIP1;2 and rice NIP sequences. A test neighbor-joining phylogenetic tree was built with the same software based on the alignment.

### Plant material and growth conditions

*Oryza sativa* L. *ssp. indica “*Minghui86” (“MH86”) served as the transformation host and the wild-type (WT) control. Rice seeds were sterilized for 15–20 min with 20% bleach, then germinated at 30°C for 5 days in the dark. The seedlings with uniform growth were selected and transferred to the control (-Al) growth solution (pH 4.2) overnight to adapt to the low pH condition. Then 10 seedlings for each treatment were transferred to hydroponic growth solutions containing different concentrations of AlCl_3_ (10, 20, 40, 60, 80, 120, 160 μM) with the light/temperature condition of 14 h day/10 h night at 30/25°C (day/night). The growth solution contained the macronutrients (mM): KCl, 1.0; NH_4_NO_4_, 1.5; CaCl_2_, 1.0; KH_2_PO_4_, 0.045; MgSO_4_, 0.2; MgNO_3_, 0.5; MgCl_2_, 0.155; and the micronutrients (μM): MnCl_4_, 11.8; H_3_BO_3_, 33.0; CuSO_4_, 0.8; ZnSO_4_, 3.06; Na_2_MoO_4_, 1.07; Fe-HEDTA,77.0. The pH of the solutions was maintained at pH 4.2 by 2 mM Homo-PIPES (homopiperazine-N, N’-bis-2-ethanesulfonic acid). Representative rice seedlings were chosen for root growth phenotyping and image taking after 7-days growth in a hydroponic solution (pH 4.2) containing 0 or 60 μM AlCl_3_. Root growth was determined by measuring the root length of individual seedlings prior to and after the 7-day Al treatment (50 μM). Relative root growth (RRG%) was expressed as the root growth of individual plants under Al treatment (+Al) normalized to the average root growth under the control condition (-Al).

For testing rice plants’ responses to other metal toxicity, WT (cv. MH86, *Indica*) and rice plants were treated for 24 h in growth solutions (pH 4.2) containing (in μM) CdCl_2_, 20; ZnSO_4_, 100; AlCl_3_, 50; or LaCl_3_, 5 (Huang et al., 2012). Then, the root growth of the primary roots of individual lines was determined.

### Construct preparation and rice transformation

The CRISPR-P program was used to predict the two target sites with the PAM (protospacer adjacent motifs) sequences in the exon I and II of *OsNIP1;2* (Lei et al., 2014). Subsequently, single guide RNA (sgRNA) sequences targeted the selected *OsNIP1;2* sequences were cloned into the vector VK005-01, which contains a maize ubiquitin promoter for Cas9 expression and a rice U6 promoter for sgRNA expression (Viewsolid Biotech, Beijing, China). The primer sequences for the two *OsNIP1-2* targets are sgRNA1F: 5′-CAGTGGTCCAAGGAGGCCGTCGT-3′ and sgRNA1R: 5′-A CACGACGGCCTCCTTGGACCA-3′; sgRNA2F: 5′-CAGACC CTGCCTGCTGAAGAACG-3′ and sgRNA2R: 5′-AAC CGTT CTTCAGCAGGCAGGGT-3′. The constructs were transformed into *Agrobacterium tumefaciens* (EHA105) and then introduced into rice calli of “MH86” with the previously reported methods (Hiei et al., 1994).

### Genotypic analysis for rice CRISPR-Cas9 lines

Leaf genomic DNAs were extracted with CTAB (cetyltrimethylammonium bromide) (Stewart, 1993). The Cas9/sgRNA T-DNA insertion in the transgenic plants was examined by PCR amplification with the following primer pairs, Cas9F: 5′-GGGAGATCCAGCTAGAGGTC-3′ and Cas9R: 5′-GGAAGGAGGAAGACAAGG-3′. The sgRNA target region of *OsNIP1;2* was amplified with the following target-specific primer pairs (OsNIP1-2T1F/R and OaNIP1-2T2F/R), OsNIP1-2T1F: 5′-TGCCGAAGCCTGCTGCTTTC-3′ and OsNIP1-2T 1R: 5′-CGCAAGTTTGGCAAACCACTTG-3′, OsNIP1-2T2F: 5′-GATGGCGGTGGTGGTCGAC-3′, and OsNIP1-2T2R: 5′-G ACTCAATCAGAACACGGTTG-3′, respectively. The PCR products were sequenced. The resulting sequences were decoded with a decoding tool (Liu et al., 2015), and the mutation types and frequency were analyzed. DNA sequences were aligned with the MEGA 6.06 software.

### RNA preparation and gene expression quantification

Seedlings of the WT (*indica*) and *Osnip1;2-1*, *Osnip1;2-2* mutants were treated with various Al concentrations (0–120 μM) for the indicated duration or different metals. Root tips (0–3 cm) and other tissues (root, stem, leaf, panicle, or seed) were collected in liquid N2 and stored at −80*^o^*C.

Total RNAs were extracted with an RNeasy Mini Kit [QIAGEN China (Shanghai) Co., Ltd., Shanghai, China] and used to synthesize first-strand cDNAs with the SuperScript III First-Strand Synthesis System [Invitrogen China (Shanghai) Co., Ltd., Shanghai, China].

*OsNIP1;2* expression was investigated by RT-qPCR with a 7500 Fast Real-Time PCR System (Applied Biosystems). The expression of target genes was calibrated with an endogenous 18S rRNA. The RT-qPCR primer sequences for *OsNIP1;2* are 5′-CTCCTTCTTCCTCATGTTCG-3′ and 5′-CCTGCGAA GAGCACGTTCA-3′.

### Subcellular localization of OsNIP1;2 in plant cells

The coding sequence of *OsNIP1;2* without the stop codon was amplified from a cDNA plasmid using the primers 5′-TCGCGGATCCAAA ATGGCGGTGGTGGTCGAC-3′ and 5′ -ATGGCTCGAG ACTCCTACGCGAGCTCCTC-3′ (the underlined sequences represent the *Bam*HI and *Xho*I cutting sites, respectively). The PCR sequences were then cloned into the *pGPTV.GFP.Bar* vector in front of the GFP coding sequence. The resultant *pGPTV-OsNIP1;2-GFP* construct was introduced into *A. tumefaciens* strain GV3101 and then transiently expressed in tobacco leaf epidermal cells with an infiltration method. The plasma membrane marker *35S:PIP2;1-RFP* (pm-rk-CD3-1007) was described previously (Nelson et al., 2007). The fluorescent images were obtained with a Leica SP5 confocal scanning laser microscope. The nuclei of the tobacco leaf cells were stained with DAPI (4′,6′-diamidino-2-phenylindole) and visualized with the confocal microscope.

### OsNIP1;2 expression, localization, and function in yeast

#### Al sensitivity assays

For Al sensitivity evaluation, the *pYES2-OsNIP1;2* construct was generated with *Hind* III and *Xba*I double enzyme digestion of the *pYES2* vector, and the PCR amplified product from the *OsNIP1;2* cDNA with primers 5′-TCGCAAGCTTAAAATGGCGGTGGTGGTCGAC-3′ and 5′- ATCCTCTAGACTAACTCCTACGCGAGCTCC -3′ (the underlined were *Hind*III and *Xba*I restriction sites, respectively). The resulting *pYES2-OsNIP1;2* construct and the *pYES2* empty vector were introduced into a wild-type yeast strain (BY4741). Three independent colonies from each transformation event were selected to represent 3 biological replicates for the following experiments.

Individual yeast colonies were grown in a liquid SD-Ura medium to the stationary phase. Yeast cells were collected by centrifuging (5,000 × *g*) for 5 min and washed three times with a succinic acid-buffered LPM (low-pH, low-magnesium) medium (pH 4.2). The washed cells were grown at 30°C in a new LPM medium containing 2% galactose for GAL promoter induction. After 6-h growth, the yeast cells were collected by centrifuge at 5,000 *g* for 5 min. Then, the cell pellets were diluted to OD630 = 0.2 with an LPM growth medium containing 2% galactose. Then, 10 μl of fivefold serially diluted cell suspensions were placed onto the LPM solid media (pH 4.2) containing 0, 150, or 300 μM AlCl_3_ and grew at 30°C for 3 days. LPM contained the below macronutrients (mM): KCl, 5; (NH_4_)_2_SO_4_, 40; NaCl, 2; CaCl_2_, 0.1; KH_2_PO_4_, 0.01; MgSO_4_, 0.25; and the micronutrients (μM): FeCl_3_,1; KI, 0.5; H_3_BO_3_,10; MnSO_4_, 2.5; Na_2_MoO_4_, 1; ZnSO_4_, 1.5; and amino acids (mg/L): Glu, 0.075; Tyr, 0.03; Ade, 0.02; Ura, 0.02; Val, 0.15; Phe, 0.05; Ser, 0.4; Leu, 0.03; Ile, 0.03; Arg, 0.02; Lys, 0.03; Trp, 0.02; His, 0.02; Met, 0.02; Asp, 0.0625; Thr, 0.2; and 2% Galactose and Vitamins (ng/L): Riboflavin, 20; Folic acid, 0.2; p-aminobenzoic acid, 20; Biotin, 0.2; Calcium pantothenate, 40; Pyridoxine hydrochloride, 40; Niacin, 40; Inositol, 200; Thiamine hydrochloride, 40.

#### Subcellular localization of OsNIP1;2 in yeast

To detect OsNIP1;2 subcellular localization in yeast, the coding sequence of GFP protein was PCR amplified from the *pGPTV.GFP.Bar* vector with primers 5′-ATCCGCGGCCGCCATGAGTAAAGGAGAAGAACTTTTC-3′ and 5′-TCGCTCTAGATTTGTATAGTTCATCCATGCCATG-3′ (the *Not*I and *Xba*I cutting sites were underlined). Then the GFP sequence was sub-cloned into *pYES2* to form the *pYES2-GFP* vector. Next, the *OsNIP1;2* coding sequence was amplified from the rice cDNAs with primers 5′-TCGC GGTACCAAAATGGCGGTGGTGGTCGAC-3′ and 5′- ATCC GCGGCCGCCTAACTCCTACGCGAGCTCC-3′ (the *Kpn*I and *Not*I enzyme sites were underlined, respectively). Then, individual PCR fragments were subcloned into the *pYES2-GFP* construct, in-frame with the GFP coding sequence. Finally, the resulting *pYES2-OsNIP1;2-GFP* construct was introduced into the yeast cells, and the subcellular localization of OsNIP1;2 was observed by a confocal microscope (Leica SP5).

#### Short-term Al uptake assays in yeast

The yeast lines *pYES2-GFP* and *pYES2-OsNIP1;2-GFP* were inoculated in a liquid LPM medium (pH 4.2) and grown at 30°C to a mid-exponential phase. Then, yeast cells were harvested by centrifuge at 5,000 × *g* for 5 min and washed three times with the LPM medium. The cell pellets were resuspended in an LPM medium containing 2% galactose and grown at 30°C for 2 h to induce the GAL-promoter-driven *GFP* or *OsNIP1;2* gene expression. The pre-cultured yeast cells were resuspended at OD600 = 3.0 in the uptake medium, i.e., the LPM medium (pH 4.2) supplemented with different possible OsNIP1;2 transport substrates at final concentrations of 0 or 50 μM AlCl_3_: 150 μM ligands. The Al-ligands tested included 0 or 50 μM AlCl_3_, Al-Malate, Al-Citrate, Al-oxalate, Al-succinate, Al-fumarate, Al-aconite, Al-cysteine, Al-histidine, Al-glutathione, Al-phytochelatin, Al-metallothionein.

After incubation in the uptake media for 4 h, yeast cells were harvested by centrifuge (5,000 × *g*) and washed three times with deionized water (ddH_2_O) (MilliQ; Millipore). The cell pellets were dried in a 55°C oven for 2 days. The Al concentrations of each digested sample were determined by inductively coupled plasma mass spectrometry (ICP-MS) using an Agilent 7500 Series ICP mass spectrometer. Three biological replicates for each line and each treatment were conducted.

### Measurement of Al concentrations in the roots and shoots

For quantifying Al and other elements, 5-days-old seedlings of the WT (*indica*), *Osnip1;2-1*, and *Osnip1;2-2* were grown in a growth medium (pH 4.2) containing 50 or 100 μM Al for 8 h. Then, the seedlings were rinsed with a 0.5 mM CaCl_2_ solution three times and ddH_2_O twice. Shoot and root samples were collected, dried, and digested with pure HNO_3_. ICP-AES (inductively coupled plasma-atomic emission spectrometry, Thermo Fisher, iCAP 7500 Series) was used to determine the mineral elements of the samples. Each treatment contained three biological replicates.

### Determining Al concentrations in root cell sap and root cell wall

Seedlings (5-days-old) were grown in an LPM medium (pH 4.2) containing 50 or 100 μM Al for 8 h. Then, the root apices (0–3 cm) were cut and washed three times with 0.5 mM CaCl_2_ and twice with ddH_2_O. Next, the apoplastic solution of the root segments was removed *via* centrifuging at 3,000 *g* and 4°C for 10 min with the Ultra free-MC Centrifugal filter unit (Millipore) (Xia et al., 2010). Then the samples retained in the filter units were sored at -80°C. To separate the root cell sap from the root cell wall, the frozen samples with the filter units were thawed at 37°C and then centrifuged at 20,600 × *g* for 10 min. The cell-sap samples were collected from the centrifuge tubes while the cell-wall samples remained in the filter units. The root-cell-wall samples were washed three times with 70% ethanol to remove membrane fractions and then digested with 1 mL of 2 N HCl with gentle shaking for 24 h. Al concentrations were measured by ICP-AES.

### Analyzing K and Al concentrations in xylem sap

Five seedlings (5-days-old) of the *Osnip1;2-1* and *Osnip1;2-2* mutants and the WT (*indica*) were pre-treated in an LPM medium (pH 4.2) containing 50 or 100 μM Al for 8 h. Then, plants’ culms were removed, and the xylem-sap exudates were collected in a high humidity environment, with the first droplets excluded to avoid contamination. A micropipette was used to measure the volumes of the collected xylem-sap samples. The unit volume’s K and Al concentrations of the samples were determined by ICP-MS.

## Results

### Sequence alignment, phylogenetic analysis, and isolation of *Osnip1;2* mutants

There are 10 NIP members in the rice genome (*Oryza sativa*) (Bansal and Sankararamakrishnan, 2007). Phylogenetic analysis (Supplementary Figure 1) and sequence alignment (Supplementary Figure 2) indicated that AtNIP1;2 and its closest homolog, OsNIP1;2, share 63.7% amino-acid sequence identity.

Aquaporins (AQPs) share highly conserved structural features (Hove and Bhave, 2011), containing two highly conserved constrictions in the pore region thought to specialize AQPs’ functions (de Groot and Grubmuller, 2001; Forrest and Bhave, 2007). The first constriction consists of two highly conserved NPA (asparagine-proline-alanine) motifs in the inter-helical loop B (LB) and loop E (LE) (Supplementary Figure 2). The second constriction is the so-call ar/R (aromatic/arginine) region, which contains four residues located in helix 2 (H2), H5, LE1, and LE2 (Supplementary Figure 2; de Groot and Grubmuller, 2001; Wallace and Roberts, 2004; Forrest and Bhave, 2007).

The plant’s unique NIP members could be further classified into NIP-I and NIP-II subgroups (Wallace and Roberts, 2004; Wallace et al., 2006). Members of the NIP-I subgroup have a conserved ar/R tetrad sequence of Trp (W), Val/Ile (V/I), Ala (A), and Arg (R), and an invariable NPA triad sequence for the NPA1motif. However, for the NPA2 motifs, the triad sequence of a NIP-I member could be variable as NPA, NPG, or NPV (Mitani et al., 2008). OsNIP1;2 and AtNIP1;2 are NIP-I members and share a conserved ar/R tetrad sequence of WVAR (Supplementary Figure 2). However, they have different triad sequences in the NIP2 motif, i.e., NPG in AtNIP1;1 and NPA in OsNIP1;2 (Supplementary Figure 2).

To evaluate the biological function of OsNIP1;2 in rice, a CRISPR/Cas9 system was used to generate *Osnip1;2* mutants. Through *Agrobacterium*-mediated genetic transformation, 27 tissue-cultured plantlets were obtained, among which 25 plantlets showed edited *OsNIP1;2* sequences, indicating an editing efficiency of 92.6%.

Four types of plantlets could be identified by sequence decoding in the T_0_ generation, including those with homozygous, monoallelic heterozygous, biallelic heterozygous, and non-editing. First, an online CRISPR-P tool was used to screen five potential off-target sites carrying three to five mismatched bases^1^ (Supplementary Table 1). No off-target effects were identified in selected putative loci against sgRNA1 and sgRNA2 (Supplementary Table 1). Then, two homozygous mutant lines, with one nucleotide deletion in target 1 or one nucleotide insertion in target 2, which led to the shift of open reading frame and mutated *OsNIP1;2*, were designated as *Osnip1;2-1* and *Osnip1;2-2* (Supplementary Figures 3, 4) and chosen for further characterization.

### Phenotypic analysis of *Osnip1;2* mutant lines

**FIGURE 1:**
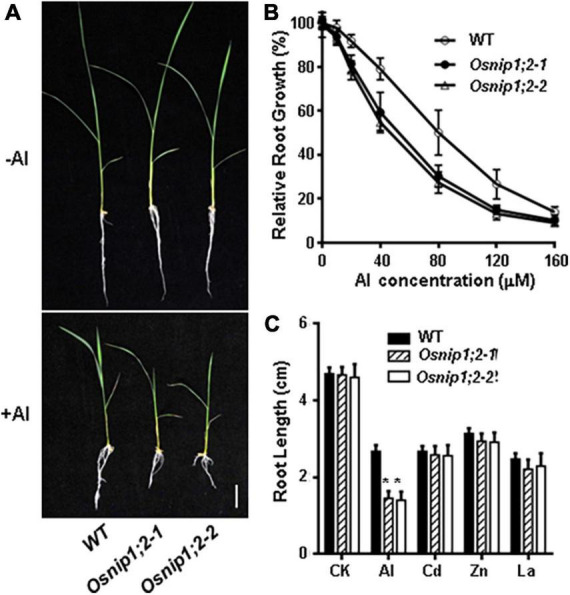
Mutations in *OsNIP1;2* sensitize the rice mutants to Al stress. **(A)** Growth phenotypes of the WT [*Oryza sativa* L. *ssp. Indica “*Minghui86” (“MH86”)] and two *Osnip1;2* mutant lines under Al stresses. Seedlings were grown in a hydroponic solution (pH 4.2) containing 0 or 60 μM AlCl_3_ for 7 days. **(B)** Relative root growth of WT and *Osnip1;2* mutants treated with different Al concentrations for 2 days. **(C)** Root length (cm) of the WT and two *Osnip1;2* mutant lines under various metal stresses (50 μM AlCl_3_; 20 μM CdCl_2_, 100 μM ZnSO_4_, or 5 μM LaCl_3_ for 24 h. Data in panels **(B,C)** are mean ± SD of 10 biological replicates. Scale bar = 1 cm. Asterisks indicate significant differences between WT and *Osnip1;2* mutants (*P < 0.05).

Root growth of the wild-type (WT), *Osnip1;2-1*, and *Osnip1;2-2* plants was comparable under the -Al (control) condition (Figure 1A). However, root growth of *Osnip1;2-1* and *Osnip1;2-2* showed more potent root-growth inhibition by 500 μM Al than the WT (Figure 1A). Moreover, such a root-growth inhibition in the two *Osnip1;2* mutant lines were Al-dose dependent (Figures 1A,B), indicating that OsNIP1;2 plays a crucial role in Al tolerance in rice.

The responses of the *Osnip1;2* mutants to toxic levels of other metal ions, including La^3+^, Zn^2+^, and Cd^2+^, were examined to investigate the sensitive specificity to Al stress. The results indicated that the WT and mutants showed no difference in growth inhibition by 20 μM CdCl_2_, 5 μM LaCl_3_, or 60 μM ZnSO_4_ (Figure 1C). Therefore, the *Osnip1;2* mutants are specifically sensitive to Al toxicity.

### *OsNIP1;2* expression is induced by Al stress

**FIGURE 2:**
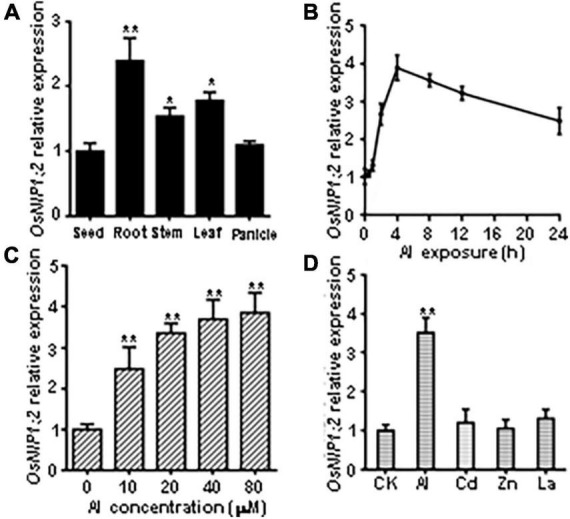
Patterns of *OsNIP1;2* gene expression in rice. **(A)** Detection of *OsNIP1;2* expression in the seed, root, stem, leaf, and panicle by RT-qPCR analysis. **(B)** Time-course RT-qPCR analysis of *OsNIP1;2* expression in the WT roots exposed to 50 μM AlCl_3_. **(C)** *OsNIP1;2* expression in the WT root treated with different Al concentrations. **(D)** RT-qPCR analysis of *OsNIP1;2* expression in the WT roots in response to different metal ions. The WT plants (5-days-old) were treated for 6 h with 50 μM AlCl_3_; 20 μM CdCl_2_; 5 μM LaCl_3_; or 100 μM ZnSO_4_. Asterisks indicate significant differences between CK and AlCl_3_ treatments (**P < 0.01).

RT-qPCR analyses showed *OsNIP1;2* transcript levels were significantly higher in the root, stem, and leaf than in the seed and panicle (Figure 2A). Time-course RT-qPCR analyses indicated that *OsNIP1;2* transcripts were rapidly up-regulated in the root, peaked at 4 h after Al treatment, and remained high after 24 h (Figure 2B). Furthermore, increased Al concentrations in the treatment solutions were associated with enhanced *OsNIP1;2* expression in the root (Figure 2C). In addition, *OsNIP1;2* expression was induced by Al^3+^ ions but not responsive to Zn^2+^, Cd^2+^, and La^3+^ (Figure 2D).

### OsNIP1;2 is localized to the plasma membrane

**FIGURE 3:**
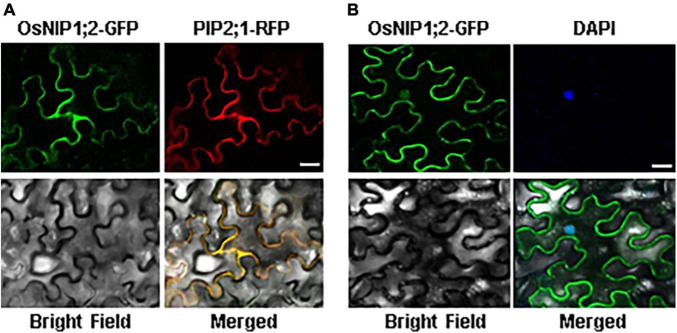
The OsNIP1;2 protein was localized to the plasma membrane of *Nicotiana benthamiana* epidermal cells. **(A)** The OsNIP1;2-GFP (green) fusion protein was colocalized with the PM marker PIP1;2:RFP (red) when transiently expressed in the tobacco epidermal cell. **(B)** The nucleus (blue) of a tobacco epidermal cell expressing 35S:OsNIP1;2:GFP was stained with DAPI. The image was observed under a confocal laser microscope. Scale bar: 10 μm.

The subcellular localization of OsNIP1;2-GFP was evaluated by transient co-expression of OsNIP1;2-GFP and the red fluorescence protein (RFP)-PIP2;1, a PM marker protein, in tobacco (*Nicotiana benthamiana*) leaf epidermal cells (Nelson et al., 2007). The RFP-PIP2;1 signal was localized in the plasma membrane of *N. benthamiana* cells, overlapping with OsNIP1;2:GFP (Figure 3A). In addition, the DAPI-stained nucleus was enclosed by the OsNIP1;2-GFP fluorescence in the cytoplasm (Figure 3B). These results demonstrated OsNIP1;2 as a PM-localized protein.

### OsNIP1;2 affects Al distribution in rice

**FIGURE 4:**
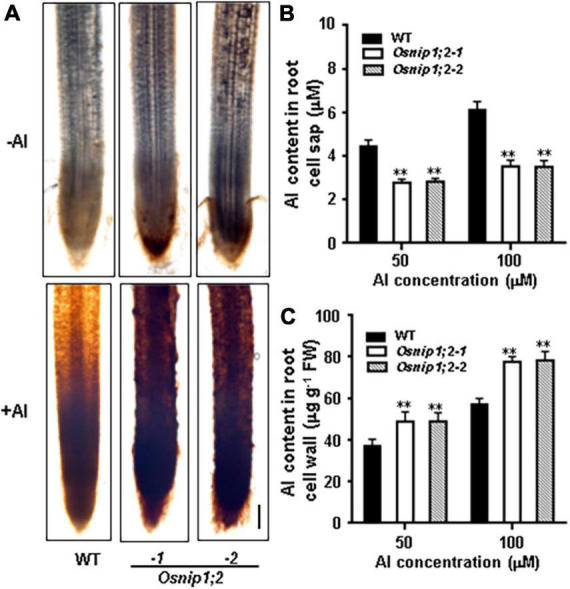
Aluminum distribution in rice root. **(A)** Hematoxylin staining indicated aluminum accumulation in the WT and *Osnip1;2* mutants’ root cell walls. Scale bar, 100 μm. **(B)** The Al concentration in the root cell sap. **(C)** The Al concentration in the root cell wall. WT, *Osnip1;2-1*, and *Osnip1;2-2* plants (5-days-old) were exposed to 50 or 100 μM AlCl_3_ (pH 4.2) for 8 h. The root-cell-sap Al concentrations **(B)** and the root-cell-wall Al contents **(C)** were determined by ICP-AES. Data are the average of three biological replicates. ^**^P < 0.01 between WT and individual *Osnip1;2*. FW, fresh weight.

Al accumulation in the root cell wall can be visualized by hematoxylin staining. When the roots of WT, *Osnip1;2-1*, and *Osnip1;2-2* seedlings were stained with hematoxylin after Al treatment, the *Osnip1;2-1* and *Osnip1;2-2* plants showed much stronger hematoxylin staining in the root apex than the WT plants (Figure 4A). These results suggest that OsNIP1;2 facilitates Al removal from the root cell wall.

Furthermore, compared with the WT plants, the Al-treated *Osnip1;2* mutants had significantly lower Al concentrations in the root cell sap (Figure 4B). In contrast, the root-cell-wall Al concentrations were considerably higher in the *Osnip1;2* mutants than in WT plants (Figure 4C). These results indicate that OsNIP1;2 participates in removing the Al in the root cell wall to the cytosol.

**FIGURE 5:**
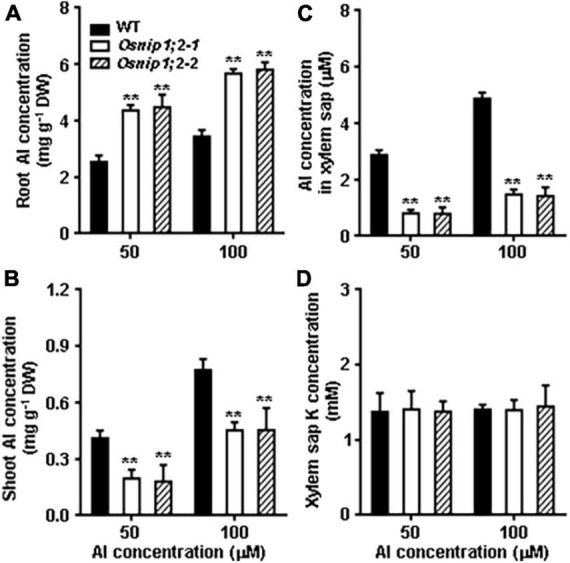
Al distribution in Rice. Total Al concentrations in the root **(A)**, shoot **(B)**, xylem sap **(C)**, and the K concentration in the xylem sap **(D)**. Plants were grown in the nutrient solution for 5 days and then exposed to 50 or 100 μM AlCl_3_ for 8 h. Then, Al concentrations were measured using ICP- AES. Data are the average of three biological replicates. **P < 0.01 between WT and individual *Osnip1;2* lines. DW, dry weight.

We measured Al concentrations of the root and shoot after Al treatment to further evaluate the role of OsNIP1;2 in the root-to-shoot Al distributions. Compared with the WT, the *Osnip1;2-1* and *Osnip1;2-2* mutants possessed remarkably higher and lower Al concentrations in the root (Figure 5A) and shoot, respectively (Figure 5B). These results indicated that OsNIP1;2 plays a vital role in Al translocation to the shoot in rice.

The role of OsNIP1;2 in loading Al to the xylem was examined by evaluating Al concentrations in the root xylem exudates. The results indicated that Al concentrations of the root xylem exudates were considerably lower in the *Osnip1;2-1* and *Osnip1;2-2* mutants than in the WT (Figure 5C), while the K concentrations were comparable among the WT and the *Osnip1;2* mutants (Figure 5D). Furthermore, compared to the WT, 43 and 68% decreases in Al concentrations were observed in the root cell sap (Figure 4A) and the root xylem exudate (Figure 5C), respectively, in the *Osnip1;2* mutants. The extra percentage decreases in Al concentrations in the xylem sap of the *Osnip1;2* mutants could be attributed to the impaired function of OsNIP1;2 in loading Al to the xylem of the root cells.

Taken together, our results suggested that OsNIP1;2 involves removing Al from the root cell wall and promotes Al translocation to the shoot by facilitating Al loading to the root xylem.

### Decreased Al tolerance by heterologous expression of OsNIP1;2 in yeast

**FIGURE 6:**
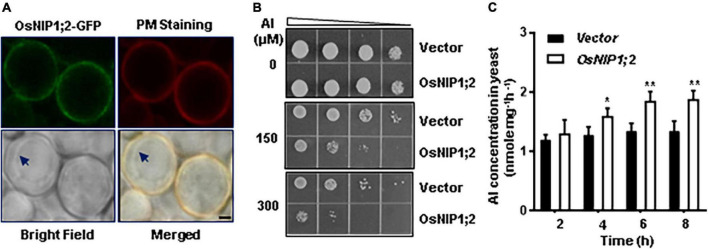
Functional analysis of OsNIP1;2 in yeast. **(A)** Subcellular localization of OsNIP1;2 in yeast. The yeast cells expressing the OsNIP1;2:GFP fusion protein (green) were stained with the red CellMask Plasma Membrane Staining reagent (Thermo Fisher Scientific) and observed under a confocal laser microscope (Scale bar: 1 μm.). Arrows point to the tonoplast. **(B)** Ten microliters of yeast cell suspension with an OD of 0.2 and four serial 1:5 dilutions were spotted on LPM agar plates supplemented with 0, 150, or 300 μM AlCl_3_. The *pYES2-GFP* (GFP) or the *pYES2-OsNIP1;2-GFP* (OsNIP1;2-GFP) yeast lines were used for the growth experiments. **(C)** OsNIP1;2-mediated Al uptake in yeast. Gene expression of *GFP* or *NIP1;2-GFP* in yeast cells was induced by 2% galactose for 2 h. Al uptake was determined by analyzing the differences in Al contents of the AlCl_3_-treated (50 μM) yeast lines for 0–8 h. Data are the average of three biological replicates. Asterisks mark significant differences between two yeast lines (*P < 0.05, **P < 0.01).

We transferred the *pYES2-GFP* (control) and *pYES2-OsNIP1;2-GFP* (*OsNIP1;2*) constructs to the wild-type (BY4741) yeast cells. When expressed in the yeast cell, the OsNIP1;2-GFP fusion protein was detected at the plasma membrane under a confocal microscope (Figure 6A). This result was consistent with the OsNIP1;2 subcellular localization observed in *Nicotiana benthamiana* epidermal cells (Figure 3).

We compared the Al sensitivity of the transformed yeast lines to test if OsNIP1;2 could contribute to Al resistance in yeast. Without Al treatment, the *OsNIP1;2-*expressing line and the control (empty vector) line displayed similar cell-growth rates (Figure 6B). In contrast, expressing *OsNIP1;2* in yeast led to remarkably more sensitivity of the yeast line to Al stress (Figure 6B).

Short-term (up to 8 h) Al uptake by OsNIP1;2 were tested in the presence of Al^3+^ (Figure 6C) or Al^3+^ complexed with different cellular ligands, including citrate (Cit), malate (Mal), oxalate (Oxa), succinate (Suc), fumarate (Fum), aconite (Aco), cysteine (Cys), histidine (His), glutathione (GSH), phytochelatin (PC), and metallothionein (MT) (Supplementary Figure 5). In a short-term (up to 8 h) time-course assay, significant OsNIP1;2-mediated Al uptake activities were observed in the *OsNIP1;2*-expressing yeast cells after incubation in the uptake solution containing 50 μM AlCl_3_ for 4 h (Figure 6C). This result suggested that OsNIP1;2 facilitated Al uptake in yeast.

To test the effects of different cellular ligands on OsNIP1;2-mediated Al uptake, we performed a short-term (4 h) Al uptake assay for the yeast lines (BY4741) carrying the *pYES2-GFP* or *pYES2-OsNIP1;2-GFP* construct in the presence of Al^3+^ or Al^3+^ conjugated with different cellular ligands mentioned above. The *pYES2-OsNIP1;2-GFP* line showed significantly enhanced Al uptake activities in the presence of Al^3+^ and the Al-Cys conjugated complex in the uptake solution but not with other Al-ligands (Supplementary Figure 5). This result suggests that the Al-Cys complex could be a transport substrate for OsNIP1;2.

## Discussion

### OsNIP1;2 is probably an aluminum transporter

Aquaporin members can transport various small molecules, including B, As, glycerol, Al-malate complexes, and Zn complexes (Wang et al., 2017, 2022; Singh et al., 2020). OsNIP1;2 is one of the 10 members of the NIP subfamily in rice (Bansal and Sankararamakrishnan, 2007). Expressing OsNIP1;2 caused the yeast cells to be more susceptible to Al toxicity (Figure 6B). In addition, the yeast cells expressing OsNIP1;2 displayed enhanced Al uptake activities when grown in the liquid medium supplemented with AlCl_3_ or the Al-Cys complex (Figure 6C and Supplementary Figure 5). As the OsNIP1;2 is localized to the PM in the yeast cell (Figure 6A), it is reasonable to assume that OsNIP1;2 can facilitate Al transport and uptake into yeast cells.

Our previous results suggested that the aluminum-malate (Al-Mal) complex could be a transport substrate for AtNIP1;2 (Wang et al., 2017). As the AtALMT1-facilitated malate release system accounts for most of Al resistance in Arabidopsis (Hoekenga et al., 2006; Liu et al., 2009), AtNIP1;2 plays a critical complementary role in removing the Al-Mal complex from the root cell wall for Arabidopsis plants to achieve higher overall Al resistance (Wang et al., 2018, 2020).

OsNIP1;2 is the closest sequence homolog of AtNIP1;2 (Supplementary Figure 2) and is involved in Al transport, translocation, and resistance in rice (Figures 1, 4, 5). However, OsNIP1;2 appeared not to facilitate Al-Mal transport in yeast (Supplementary Figure 5). Instead, an Al-Cys transport activity was observed for the OsNIP1;2-expressing yeast line (Supplementary Figure 5). Although AtNIP1;2 and OsNIP1;2 share a high degree of sequence homology in overall sequences, especially in the ar/R region and NPA motifs (Supplementary Figure 5), a single amino acid difference in the NPA2 constriction (Supplementary Figure 5) might change the transport substrate preferences for these two transporters.

Aquaporin transporters are believed to facilitate transporting polar but non-charged small molecules. However, a significant but minor Al transport activity was also observed for the yeast line carrying OsNIP1;2 in the presence of Al^3+^ ions (Figure 6 and Supplementary Figure 5). As yeast cells secrete cellular ligands into the growth media (Kutralam-Muniasamy et al., 2015), the external ligands and Al^3+^ could form Al-ligand complexes in the uptake medium, which could be taken up by OsNIP1;2. Therefore, the Al taken up by OsNIP1;2 in the Al^3+^ condition (Figure 6 and Supplementary Figure 5) could be in the form of Al-ligands but not as Al^3+^ ions. Further studies are required to distinguish these possibilities and to identify the identity of the ligand.

### OsNIP1;2 facilitates removing aluminum from the root cell wall and promotes its translocation to the shoot

The cell walls in the root apex are a significant target of Al toxicity. The reason is that Al toxicity disrupts the root cell walls’ integrity, structure, and function in the root tip, as evidenced by the distorted and swollen cells in the root apex (Ma et al., 2004; Horst et al., 2010; Sivaguru et al., 2013). Therefore, reducing the cell wall’s Al concentrations in the root apices could alleviate Al toxicity and thus improve plants’ Al resistance.

Decreases in Al accumulation in the root cells could be achieved by lowering the binding capacity to Al *via* modifying cell-wall components, such as reducing the polysaccharide concentrations and/or increasing degrees of pectin methylation (Yang et al., 2008, 2011). Another means is to remove Al from the root cell wall for further detoxification inside the cell (Xia et al., 2010; Wang et al., 2017). The Al could be sequestered into the vacuole of the root cell or moved to xylem parenchyma for uploading to xylem for translocation to the less vulnerable shoot *via* the xylem stream. Our previous results indicate that AtNIP1;2 functions as a bi-directional channel, facilitating the Al uptake from the root cell wall and subsequent Al uploading to the xylem sap (Wang et al., 2017).

Loss-of-OsNIP1;2 functions impaired Al removal from the root cell wall (Figure 4) and subsequent Al translocation to shoot (Figure 5), which made the mutant plants susceptible to Al toxicity (Figure 1). The results indicate that the PM-localized OsNIP1;2 is a crucial component of the internal detoxification mechanism in rice.

### The roles of OsNrat1 and OsNIP1;2 in removing aluminum from the root cell wall

In plants, members of the Nramp (natural resistance-associated macrophage protein) transporters are localized to membranes of different subcellular compartments, facilitating the transport of divalent and trivalent metal ions, including Fe^2+^, Zn^2+^, Cd^2+^, Mn^2+^, As^2+^, and Al^3+^, in monocots and dicots plants (Williams et al., 2000; Mäser et al., 2001; Pittman, 2005; Ding and Cai, 2017). Recently, Nrat1 (Nramp aluminum transporter1) has been suggested to facilitate transporting the root cell walls’ Al^3+^ ions to the cytosol in rice (Xia et al., 2010; Li et al., 2014). Then, the cytosolic Al could be transported to the vacuoles of the root cells by OsALS1 or other unknown transporters.

In contrast, members of the aquaporin superfamily are channel proteins and are believed to transport polar but non-charged small solutes (Forrest and Bhave, 2007; Maurel et al., 2008). However, our recent studies suggest that some members of the aquaporin subfamilies could be involved in transporting divalent metal ions, Zn^2+^, and trivalent valent metal ions, Al^3+^, complexed with cellular ligands such as glutathione (GSH) and malate, respectively (Wang et al., 2017, 2022). The metal-ligand complexes are presumably neutrally charged; thus, they could be transport substrates for aquaporin transporters.

Under adverse conditions, plant roots secrete various metabolites into the rhizosphere. The root exudates could adjust soil pH to solubilize mineral nutrients and make them more accessible to plants; chelate toxic compounds; facilitate the formation of beneficial microbiota communities, or function as toxic compounds for pathogens (Bertin et al., 2003; Bais et al., 2006; Badri and Vivanco, 2009; Baetz and Martinoia, 2014; Zhao et al., 2019; Vives-Peris et al., 2020; Upadhyay et al., 2022). For instance, under Al toxicity, plant roots secrete various organic acids (OAs), i.e., malate, citrate, and oxalate, into the rhizosphere (Sasaki et al., 2004; Magalhaes et al., 2007; Liu et al., 2009, 2012; Matonyei et al., 2014; Kochian et al., 2015; Qiu et al., 2019). The OAs and Al^3+^ form OA-Al complexes in the rhizosphere, which are inaccessible to root cells and thus non-toxic to plants. However, our studies indicated that the OA-Al complexes retained in the root cell wall could be toxic, and they need to be removed from the root cell wall to reach a higher resistance level for plants (Wang et al., 2017, 2020). Although root OA exudation could not explain Al resistance in rice (Famoso et al., 2011), the Al complexed with other cellular ligands in the root cell wall could be harmful to the root cells and needs to be removed.

Thus, although structurally distinct, the putative Al^3+^ transporter, Nrat1, and Al-ligand transporter, NIP1;2, are functionally overlapped but complementary in the same biochemical process to clean out the Al retained in the root cell wall under the acid soil condition.

## Conclusion

In this report, we have demonstrated that the *OsNIP1;2* gene expression is explicitly induced in the rice root by Al stress. Moreover, the impaired *OsNIP1;2* caused the mutant rice plants to be more vulnerable to Al toxicity. Thus, OsNIP1;2 is required for Al resistance in rice. In addition, phenotypic observations indicated that the OsNIP1;2 facilitates Al removal from the root cell wall and subsequent redistribution to the above-ground tissues. In addition to the hypersensitive phenotypes to elevated Al concentrations in the medium, the OsNIP1;2-expressing yeast cells accumulated higher amounts of Al in the cells and were sensitive to Al toxicity. In conclusion, OsNIP1;2 reduces root cell wall’s Al concentrations and promotes Al redistribution from the root to the above-ground tissues, vital for achieving internal detoxification of Al in rice.

## Data availability statement

The original contributions presented in this study are included in the article/Supplementary material, further inquiries can be directed to the corresponding authors.

## Author contributions

YW and JL: conceptualization, data curation, funding acquisition, investigation, methodology, visualization, supervision, project administration, and writing – original draft, review and editing. SY: data curation, investigation, methodology, visualization, and validation. CL, TH, SH, QB, XJ, FX, CG, and MH: investigation, methodology, and visualization. YC: conceptualization, investigation, and methodology. All authors contributed to the article and approved the submitted version.

## Footnotes

1
http://skl.scau.edu.cn/offtarget/
