# Supplementary material for: The plasma membrane-localized OsNIP1;2 mediates internal aluminum detoxification in rice
Source: Front Plant Sci. 2022 Sep 12;13:970270. doi: 10.3389/fpls.2022.970270 (PMC9512054; doi:10.3389/fpls.2022.970270)
Supplement: Supplementary file 1 [file Table_1.DOCX]

**Table S1.** Mutations detection on five most likely putative off-target sites.

| **Target** | **NOPO** | **Putative off-target locus** | **Putative off-target sequence** | **MMB** | **NPOM** | **OTS** | **Region** |
| --- | --- | --- | --- | --- | --- | --- | --- |
| T1 | OT1 | LOC_Os04G0531400 | GCGACCTCCGCCTTGGACGA  CGG | 5 | 0 | 0.024 | CDS |
|  | OT2 | LOC_Os02G0230625 | AGGACGGCCTCCTCGGCACA CGG | 4 | 0 | 0.019 | CDS |
|  | OT3 | LOC_Os04G0436100 | CCGACGACCTCCTTGGACAA  GGC | 5 | 0 | 0.008 | CDS |
|  | OT4 | LOC_Os05G0581100 | ACGTCGGCCTCCGAGGACCT CGG | 4 | 0 | 0.001 | CDS |
|  | OT5 | LOC_Os07G0534400 | CCGACGGCGACGTTGGACGA CGG | 5 | 0 | 0.025 | 5’ UTR |
| T2 | OT6 | LOC_Os07G0683600 | CGTTCGTCTGCACGCAGGTT  AGG | 4 | 0 | 0.096 | Intron |
|  | OT7 | LOC_Os09G0567700 | CGTTCTGCAGGAGACATGAT TGG | 5 | 0 | 0.020 | CDS |
|  | OT8 | LOC_Os12G0613250 | TGTTCTTCATCAGACAGGGT  AGC | 4 | 0 | 0.014 | CDS |
|  | OT9 | LOC_Os05G0311600 | CGTTGGTCAGCAGGGAGTGT TGG | 4 | 0 | 0.011 | CDS |
|  | OT10 | LOC_Os04G0652900 | CGTTCTTCAGCAGCCAATGT  GGT | 4 | 0 | 0.004 | 3’ UTR |

The protospacer adjacent motif (PAM) (NGG) is shown in green background. **Mismatch nucleotides are marked in red**.

NOPO; name of putative off-target, MMB; mis-matching bases, NPOM; number of plants with off-target mutations,

OTS; off-target score. T1 and T2 represent target1 and target2.
