# Supplementary figures and images for: The plasma membrane-localized OsNIP1;2 mediates internal aluminum detoxification in rice
Source: Front Plant Sci. 2022 Sep 12;13:970270. doi: 10.3389/fpls.2022.970270 (PMC9512054; doi:10.3389/fpls.2022.970270)

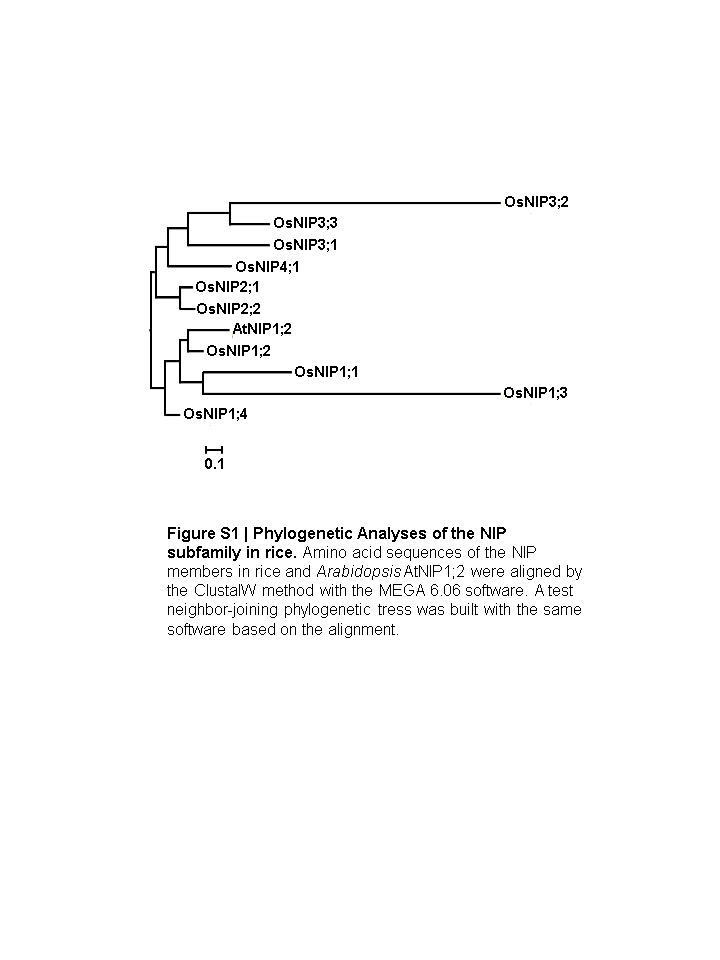

Supplement: Supplementary file 2 [file Image_1.TIF]

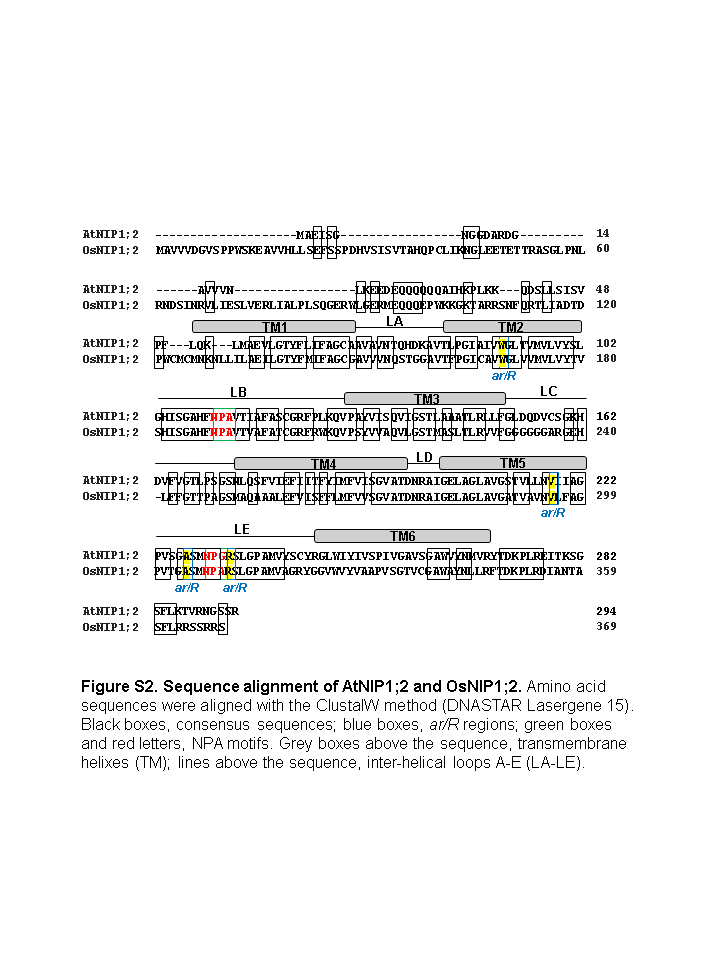

Supplement: Supplementary file 3 [file Image_2.TIF]

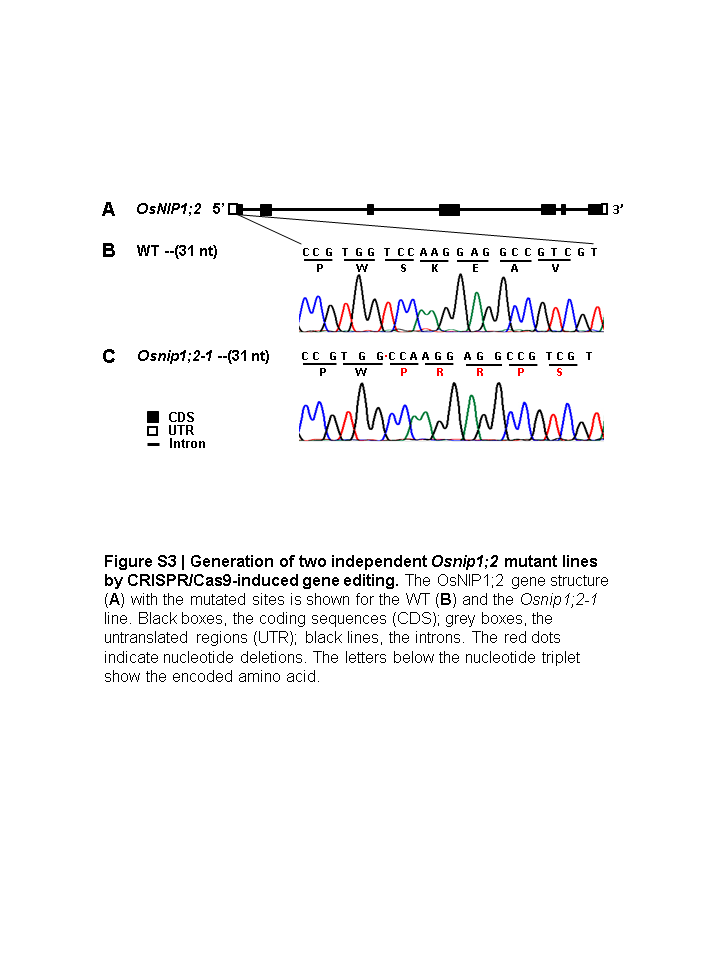

Supplement: Supplementary file 4 [file Image_3.TIF]

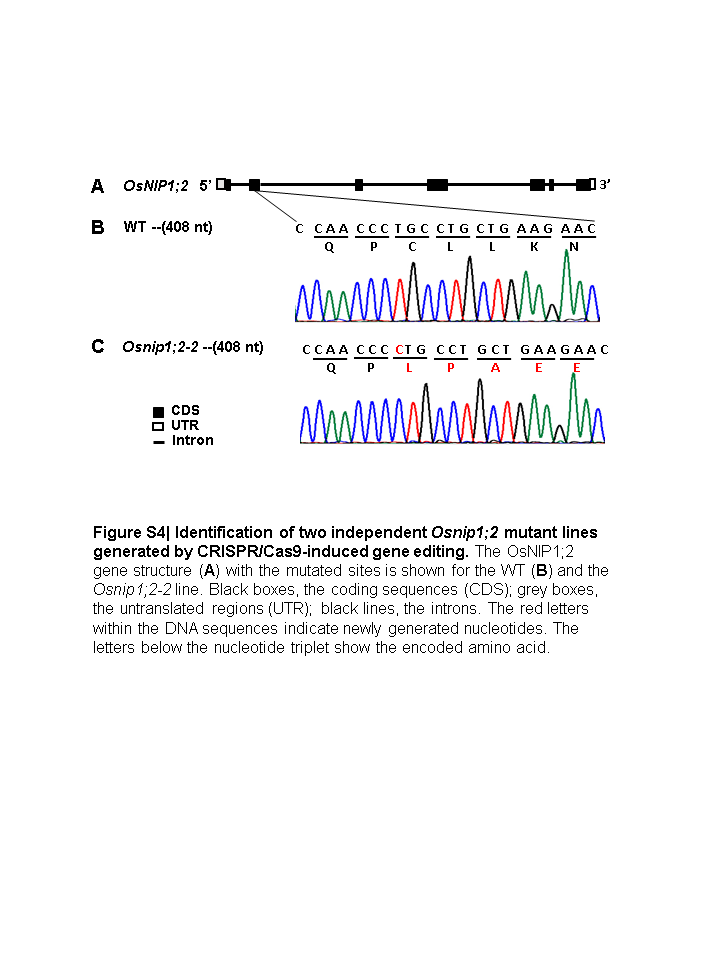

Supplement: Supplementary file 5 [file Image_4.TIF]

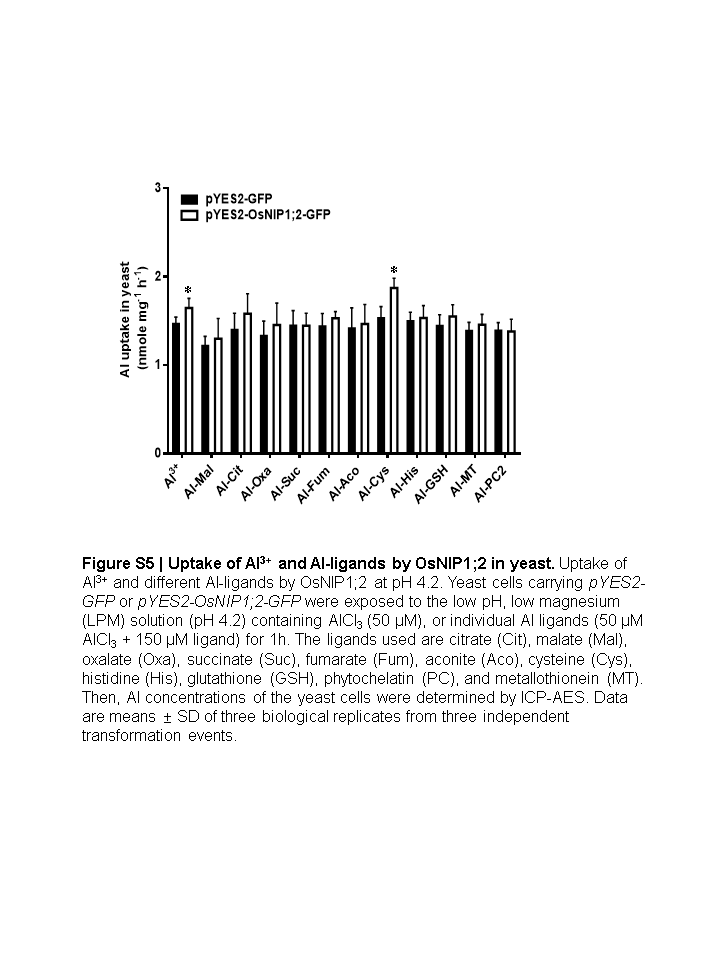

Supplement: Supplementary file 6 [file Image_5.TIF]
